# The Carbohydrate Sensitive Rat as a Model of Obesity

**DOI:** 10.1371/journal.pone.0068436

**Published:** 2013-07-30

**Authors:** Nachiket A. Nadkarni, Catherine Chaumontet, Dalila Azzout-Marniche, Julien Piedcoq, Gilles Fromentin, Daniel Tomé, Patrick C. Even

**Affiliations:** 1 Chaire Aliment, Nutrition, Comportement Alimentaire, AgroParisTech, Paris, France; 2 Unité Mixte Recherche 914, Nutrition Physiology and Ingestive Behavior, AgroParisTech, Institut Nationale de Recherche, Agronomique, Paris, France; Oklahoma State University, United States of America

## Abstract

**Background:**

Sensitivity to obesity is highly variable in humans, and rats fed a high fat diet (HFD) are used as a model of this inhomogeneity. Energy expenditure components (basal metabolism, thermic effect of feeding, activity) and variations in substrate partitioning are possible factors underlying the variability. Unfortunately, in rats as in humans, results have often been inconclusive and measurements usually made after obesity onset, obscuring if metabolism was a cause or consequence. Additionally, the role of high carbohydrate diet (HCD) has seldom been studied.

**Methodology/Findings:**

Rats (n=24) were fed for 3 weeks on HCD and then 3 weeks on HFD. Body composition was tracked by MRI and compared to energy expenditure components measured prior to obesity. Results: 1) under HFD, as expected, by adiposity rats were variable enough to be separable into relatively fat resistant (FR) and sensitive (FS) groups, 2) under HCD, and again by adiposity, rats were also variable enough to be separable into carbohydrate resistant (CR) and sensitive (CS) groups, the normal body weight of CS rats hiding viscerally-biased fat accumulation, 3) HCD adiposity sensitivity was not related to that under HFD, and both HCD and HFD adiposity sensitivities were not related to energy expenditure components (BMR, TEF, activity cost), and 4) only carbohydrate to fat partitioning in response to an HCD test meal was related to HCD-induced adiposity.

**Conclusions/Significance:**

The rat model of human obesity is based on substantial variance in adiposity gains under HFD (FR/FS model). Here, since we also found this phenomenon under HCD, where it was also linked to an identifiable metabolic difference, we should consider the existence of another model: the carbohydrate resistant (CR) or sensitive (CS) rat. This new model is potentially complementary to the FR/FS model due to relatively greater visceral fat accumulation on a low fat high carbohydrate diet.

## Introduction

Rats, and humans, exhibit variability in their individual body fat gains in response to diets of varying macronutrient composition [1,2]. These differences have been noted particularly in the case of the sensitivity to high fat diets, but in addition to “fat sensitive” rats (FS, as opposed to fat resistant, FR) that gain excessive weight and fat under a high fat diet (HFD), some “carbohydrate sensitive” (CS, as opposed to carbohydrate resistant, CR) rats have also been shown to accumulate excessive fat under a high carbohydrate diet (HCD) [3].

Differences in the sensitivity of body fat gain to macronutrient composition have been tentatively linked to various defects in the components of energy expenditure (EE), including basal metabolic rate (BMR), thermic effect of feeding (TEF), respiratory quotient (RQ), metabolic flexibility of muscles and the level of uncoupling in muscular contraction [4]; [5,6]. However, several studies have suggested that a low EE does not necessarily favor adiposity [7,8] due to variability in the results, differences in the experimental procedures, and also because numerous reports concern subjects that are already obese or overweight (raising the question of whether the observed differences are the cause or the consequence of the obesity). Alternatively, analysis of the metabolic and behavioral characteristics of FS and CS rats showed that several behavioral traits are potential predictors of their FS or CS status [3].

The goal of the present study was to investigate potential early predisposing factors to dietary obesity. We measured, during low-fat feeding, before any significant adiposity could develop and using a fasting-refeeding indirect calorimetry procedure, several components of total EE that are suspected of being linked to the predisposition to dietary obesity, including BMR, TEF and substrate partitioning (as derived from RQ). These would then be compared to longitudinal body composition measured by magnetic resonance imaging (MRI) during 3 weeks of HCD then 3 weeks of HFD.

The results validated and extended the previously described CR/CS model as adiposity gain varied substantially under HCD. Detailed analysis of the components of total EE under low-fat feeding and in response to ingestion of a single high fat test-meal did not reveal any link with adiposity gain under HFD as well as under HCD. However, we observed that RQ responded differently between CR and CS rats to ingestion of a HC meal, with adiposity gain under HCD correlated with the amplitude of the post-meal increase in RQ.

## Materials and Methods

### Animals and housing

48 male Wistar rats (Harlan), arrival weight ~225g (range 193-252g) and age 7 weeks (according to breeder’s data), were delivered as 6 groups of 8, with groups arriving sequentially over a 9 month period. Rats recovered in the laboratory for 1 week on a synthetic high carbohydrate diet (HCD; Table 1) prior to any procedures. The protocol was approved by French ethical committee N° 11-027 for AgroParisTech. A 12: 12 hour L/D cycle (Lights on at 08:00) was maintained throughout.

**Table 1:** Macronutrient composition of the high carbohydrate (HCD) and high fat (HFD) diets.

	**HCD**	**HFD**
**Weight content (g/kg)**		
Milk proteins	140.0	170.0
Starch	622.4	436.6
Sucrose	100.3	71.1
Soy oil	40.0	225.0
Minerals	35.0	35.0
Vitamins	10.0	10.0
Cellulose	50.0	50.0
Choline	2.3	2.3
**Energy content (%)**		
Protein	14.7	14.4
Carbohydrate	75.9	42.9
Fat	9.4	42.8
**Energy density (kJ/g)**	15.95	19.82
**Food quotient**	0.946	0.847

Energy density is computed assuming 16.7 kJ/g for carbohydrate and protein and 37.7 kJ/g for fat. Food quotient is computed as content in energy of: ((Carbohydrate × 1) + (Protein × 0.825) + (Fat content × 0.7)).

### Experimental design and diets

**Figure 1:**
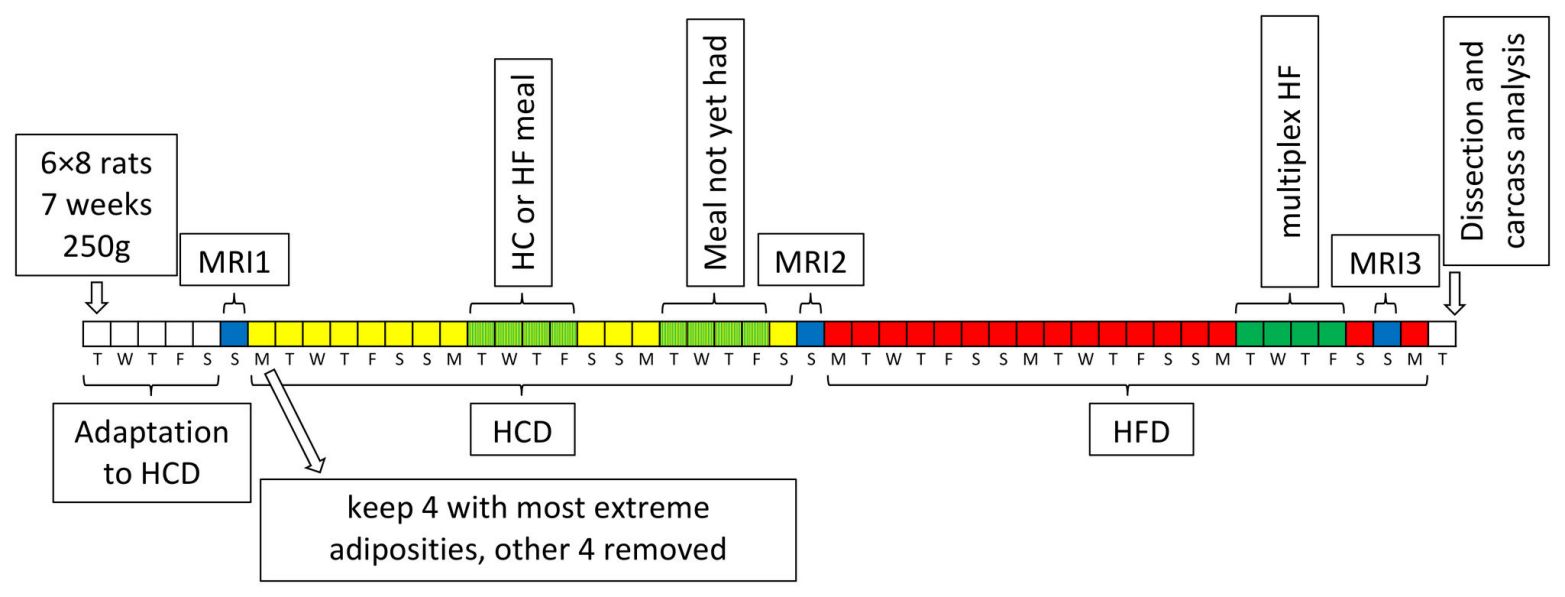
Experimental design. Blue days are MRI sessions, yellow high carbohydrate diet (HCD), red high fat diet (HFD) and green calorimetry performed during HCD. Rats arrived in groups of 8. Over the course of 9 months a total of 6 groups were processed. At arrival they were around 7 weeks of age and 225g in weight. After 1 week of adaptation to the animal housing facility and HC diet, they were processed by MRI for body composition measurement. Over the next 3 weeks of HCD, during weeks 2 and 3 on some randomly chosen day each experienced a meal-response calorimetry session, one in week 2 and the other in week 3, receiving either an HC or HF meal for the first session, and the other meal for the second session, such that each rat experienced two sessions, one with an HC meal and the other HF. Equipment was only available to analyze one rat at a time, hence the need for this arrangement, with the occupation of 4 days each week with calorimetry sessions, despite each rat individually only experiencing 1 day each week. The exact day in each week chosen for an individual and the order of HC and HF was randomized across the whole experiment. At the end of the HCD period, the rats were analyzed again by MRI, before then receiving HFD for the following 3 weeks. In the final week of the HFD period, energy expenditure was measured by indirect calorimetry with free availability of HFD (“multiplex HF”), though this data is not described here and will be reported elsewhere. Finally, there was a third MRI session and soon after the rats were sacrificed for body composition analysis and tissue harvesting.

The design of the experiment is shown in Figure 1. Rat body weight was measured every 1-2 days. The 8 rats in each group were scanned by MRI to measure their body fat content a week after arrival. Using this data, in general, for each group the 2 leanest and 2 fattest rats were kept and the other 4 discarded, though in the case of some groups it was the 3 leanest and single fattest or vice-versa that were kept if it was felt the group as a whole was particularly lean or fat. The idea was to artificially inflate the variability in starting adiposity, as one of our original intentions was to study the effect of starting adiposity on later changes in weight, metabolism and adiposity itself. The effect of this selection is shown in Figure S1.

After the first MRI measurement, the rats were fed for 3 weeks on the synthetic HCD and re-scanned by MRI at the end of this period. They were then fed for 3 weeks on a high fat diet (HFD; Table 1) prior to a third and final round of MRI. During the HCD period the rats were subjected to two calorimetry sessions at 1 week intervals and spontaneous food intake was measured towards the end of the HFD period. It should be noted that one of the calorimetry sessions during the HCD period involved consumption of a 60kJ HF meal (see details below), so there was a tiny consumption of HFD during the HCD period. At the end of the study, the rats were anesthetized with halothane, sacrificed by decapitation and blood was collected. By the third MRI session a substantial number of rats were too large to enter the MRI tunnel, or even if they could enter, their size caused image artifacts. Therefore final body composition was measured by dissection and weighing of the main organs and tissues (to the nearest 0.001g for samples <50g and 0.01g for samples >50g).

### Body composition measurement by MRI

Body composition was measured by MRI at the onset of the study and the end of the HCD period (Figure 1), plus where possible at the end of the HFD period. Images were acquired on a 7T Bruker Pharmascan system (running Paravision 4) using a Bruker 50mm i.d. tunable quadrature RF resonator. Anesthesia was induced and maintained using isoflurane in oxygen-supplemented air. Breathing rate and rectal temperature (maintained at 36-38°C using warm air) were monitored. A TurboRARE-3D sequence was used to acquire fat-sensitive T2-weighted images (TR/TE=750/42ms, FOV 75×50×50mm, matrix=128×96×96, 4-5 overlapping images acquired to cover the whole rat). Including calibration and positioning, on average each rat was unconscious for around 40 minutes. Images were registered, then fat pads segmented semi-automatically (by fuzzy c-means) in MIPAV 4.3.0. Adipose volume was converted to grams of fat mass (FM) on the assumption of a density of 0.9g cm^-3^, and fat-free mass (FFM) determined by subtracting this from the weight of the rat on the day of the scan.

### Calorimetry

During weeks 2 and 3, while fed the HCD, on a randomly chosen day each rat experienced a meal-response indirect calorimetry session, one in week 2 and the other in week 3. They randomly received either an HC or an HF meal for the first session, and the previously unchosen meal for the second session. The mean body weight of the rats was not different between HC and HF test meals (303.3±5.0g vs. 307.9±5.4g, *P*=0.53). A total of 48 calorimetry sessions were performed (24 HC and 24 HF meals). The goal of these measurements was to get from each animal values for BMR, TEF, Rest-RQ, Act-RQ and Act-cost (see next paragraph for definitions) in the fed and fasted state. For this purpose, the rats were initially housed at 18: 00 in the metabolic cages without food but with free access to water. Cage volume is 16L but the apparent dilution volume estimated from injection of a N_2_/CO_2_ mixture in the cage gives a practical dilution volume of 10.5L. The cage was ventilated at 1.5L/min. Temperature was set at 26±1°C to minimize thermoregulatory effects [9]. Between 08:00 and 10:00 the next day we measured BMR, Rest-RQ, Act-RQ and Act-cost in the post-absorptive state. At 10: 00h a calibrated HC or HF test meal (60 kJ, 3.9 g for HC, 3.0 g for HF) was provided for refeeding with the session ending around 17: 00h so that we measured TEF and evolution of Rest-RQ, Act-RQ and Act-cost in response to feeding. The size of the test meal was fixed for all the rats at 60kJ to simplify comparison of TEF between subjects (TEF varies in amplitude but also in duration as a function of meal size). Mean group body weights did not diverge by more than 7% and FFM by no more than 4%; we considered it a superior strategy to get precise estimates of TEF with these slight differences in meal size to body weight ratio rather than to try to adjust TEF for differences in meal size.

VCO_2_ and VO_2_ were measured in parallel to spontaneous activity at 5 sec intervals allowing calculation of respiratory quotient (RQ; VCO_2_ ÷ VO_2_), and via the Weir formula, energy expenditure (EE) [10,11]. The technical characteristics of the system enabled determination not only of these basic respiratory exchanges, but also those specifically associated with activity [10,12,13]. Thus it was possible to separate activity-specific EE from resting EE (REE), leading to derivation of resting metabolic rate (RMR) and the EE expended per magnitude of activity (activity cost; Act-cost). Under fasting conditions and close to thermoneutrality, RMR is **^≈^** basal metabolic rate (BMR), while post-meal increase in REE above BEE represents the thermic effect of feeding (TEF). RQ can also be separated into resting (Rest-RQ) and activity-specific (Act-RQ) components. All of these parameters were correlated to the propensity of the rats to gain adiposity during HCD and HFD. The data was binned into 15min time periods before statistical analysis. Figure S2 describes example results obtained in an individual rat.

### Adjustment of EE to body composition

The correction described in this section was only ever applied to BMR and RMR (including glucose and lipid oxidation), never to any of the other metabolic parameters where it is not needed (such as TEF, Rest-RQ, Act-RQ and Act-cost). In the case of TEF, this is because TEF is related to the size and composition of the meal, not to the size and composition of the subject [15], so TEF was computed from absolute changes in RMR post-meal vs. pre-meal (basal) RMR, not changes in RMR corrected for body size and composition.

As shown in Figure 1, meal-response indirect calorimetry sessions took place during weeks 2 and 3 of HCD thus 2-9 days away from the nearest body composition measurement. Using data from MRI1 and MRI2, and on the assumption that FFM and FM evolved linearly over this short MRI1-MRI2 period, the FFM and FM of a rat was estimated for each of the days on which it experienced an indirect calorimetry session. We controlled the validity of this extrapolation by comparing the weights estimated from MRI extrapolations of FFM+FM to actual weight measured at calorimetry and found a good agreement- according to a simple linear model the slope between predicted and actual body weight was almost 1 (0.988±0.003) with R^2^=1.000, *P*=0.000.

Since we had small sample sizes, rather than using FM and FFM directly as independent variables in the statistical models of the metabolic parameters, we decided to use normalization to FFM + 0.2 × FM [13,14]. Although results per g were calculated, they are stated per 300g to provide magnitudes similar to that of a whole rat. Because this method is not universally accepted, we also analyzed the results after adjustment to BW and to FFM only. Only the data adjusted to FFM+0.2FM are reported since all methods provided similar results, though for interest BMR adjusted to either FFM or BW is available in Table S1.

### Statistical analysis of post-processed data

Here, post-processed data means body composition values extracted from MR images, biologically meaningful parameters derived from raw calorimetry data and so on. This was all analyzed using R (version 2.15.3) [16], with graphs produced using the package ggplot2 (version 0.9.3.1) [17], linear mixed effects models calculated with the package nlme (version 3.1-108) [18], and other statistical tests carried out using standard functions.

Where used, the linear mixed effects command modeling a metabolic parameter as a function of other data took this general form:

model = lme (mp ~ adchg * mealtime, random = ~ 1 | ratID / sessionID / mealtimeID)

where lme is the linear mixed effects function called in R, mp is the metabolic parameter (RMR, TEF and so on), adchg is the percentage change in adiposity under HCD or HFD, and mealtime is pre- or post-meal. Thus four fixed effects are estimated: intercept (the metabolic parameter pre-meal in a hypothetical rat with adchg of zero), adchg (the slope describing how pre-meal the metabolic parameter is related to adiposity change), mealtime (change in the intercept after consuming a meal) and interaction (the change in the slope after the meal- meaning how response to the meal is related to adiposity change). The nested random effects are ratID (the unique identification of each rat), sessionID (each unique calorimetry session, nested within ratID) and mealtimeID (each unique pre- or post-meal period, nested within sessionID; note the distinction with the similarly-named fixed effect of mealtime defined earlier, which is the factor describing the change from the pre- to the post-meal period- so from the pre-meal mealtimeID to the post-meal one). The type of meal (HC or HF) and the type of adiposity change (under HCD or HFD) were not included in the model; the data for each of the HC and HF meals and for each of the HCD and HFD adiposity gains were analyzed separately. We also tested models where adchg was replaced with the absolute adiposity, in particular the starting adiposity measured at MRI1, as this might have an effect on metabolic parameters. However, these did not yield any interesting relationships and so are not reported.

Where and as appropriate, results are stated as mean±SEM or (only in the case of the adiposity ratios) median±0.5IQR. A large number of statistical tests were carried out for this study. Although this can sometimes justify multiple comparison correction, many of the comparisons here were of parameters that are not fully independent of each other. This makes it very difficult to carry out a fair correction so we have left the statistics uncorrected and chosen a relatively conservative threshold of *P*≤0.01 for significance, and the interval 0.01<*P*≤0.05 as marginal significance worthy of discussion.

## Results

### Body weight and adiposity evolution

**Figure 2:**
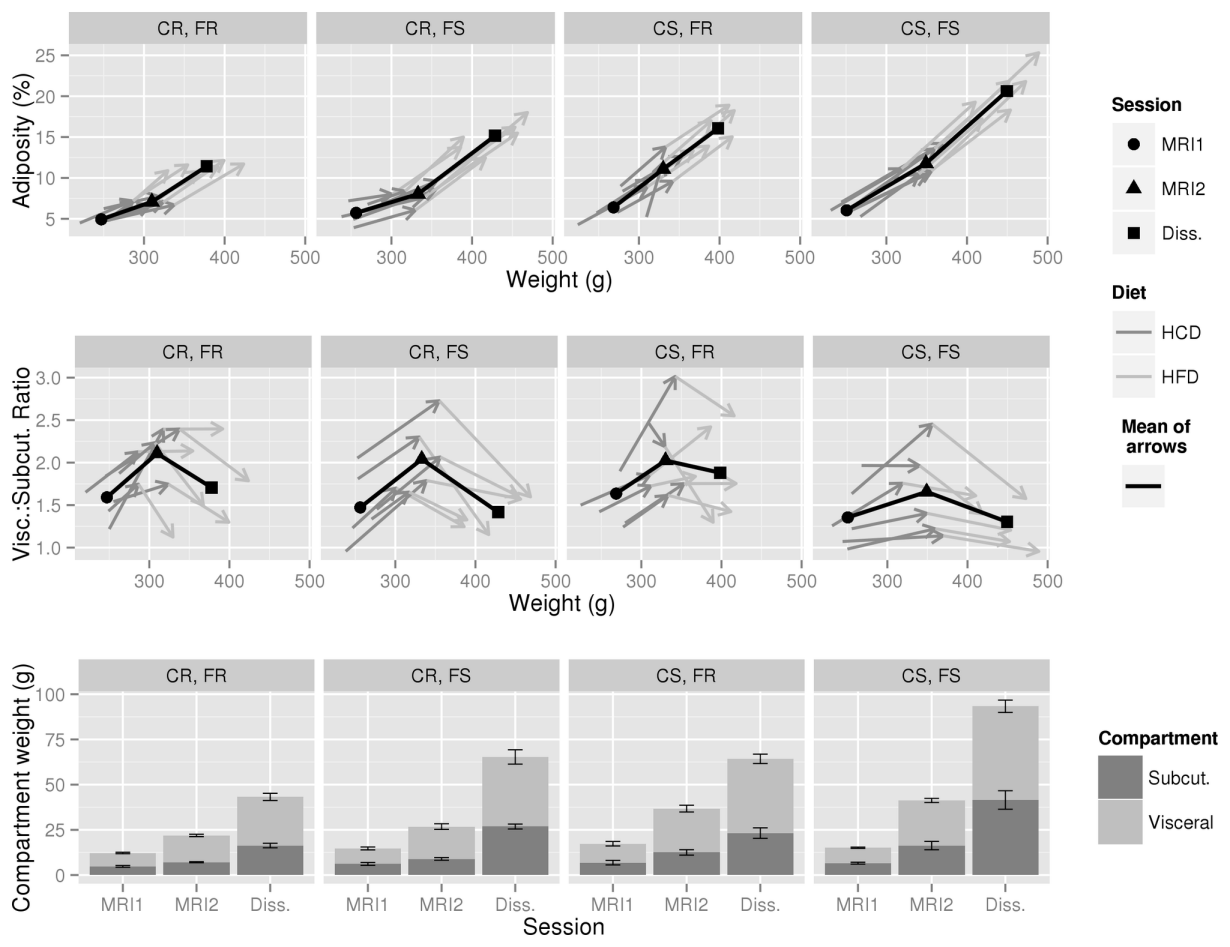
Evolution of body composition during HCD and HFD feeding. The evolution of body adiposity vs. body weight is shown in the top panel, visceral to subcutaneous fat ratio in the middle panel, and subcutaneous and visceral fat pad weights in the lower panel. The 24 rats have been sorted into groups according to whether they were in the upper (sensitive) or lower (resistant) half of rats by adiposity gain under HCD (carbohydrate resistant or sensitive; CR or CS) and/or HFD (fat resistant or sensitive FR or FS). So for example, “CS,FR” means rats that were in the upper half of adiposity gainers while under HCD, and in the lower half under HFD. In the top and middle panels, each arrow path represents a single rat, and the black point-line the mean of the arrows. In the lower panel, error bars are of group SEM. Note that each adiposity group has 6 rats; such a neat sorting was not intentional, but is instead an indication of how loose the link is between adiposity gains under HCD and HFD.

We first studied if body weight (BW) and adiposity or body fat gain correlated under high carbohydrate (HCD) and/or high fat (HFD) diets (see Figure 1 for experimental design and Table 1 for diet composition) in order to verify if, in future studies, BW gain could be used as a proxy for adiposity/fat gain. The evolution of adiposity and BW under both diets is shown in the top panel of Figure 2, and is very similar to the evolution of BW and body fat (not shown); for interest, temporal BW data is also available in Figure S3. For easier visualization, the 24 rats have been sorted into four groups depending upon whether the rat was in the lower (resistant) or upper (sensitive) half of adiposity gainers under HCD or HFD. This resistant/sensitive binary distinction is artificial, but is useful for describing the data, and is also used in the next section on calorimetry results. Thus, under HCD, the lower half of adiposity gainers have been labeled carbohydrate resistant (CR) and the upper half carbohydrate sensitive (CS). Equivalently, under HFD, the lower half are considered fat resistant (FR) and the upper half fat sensitive (FS). It should be re-emphasized here that “sensitivity” refers to rats’ gain in adiposity ((body fat ÷ BW) × 100), not gain in BW, which in itself could hide large differences in fat gain. Looking across all 24 rats (top panel, Figure 2), adiposity and BW gain tended to parallel each other more under HFD (Pearson’s r=0.911, *P*=0.000) than HCD (r=0.525, *P*=0.008), although the correlation under HCD did remain significant (this was also true when body fat gain rather than adiposity gain was taken into account). Still, the overall indication is that BW gain can be taken as a proxy of adiposity gain under a high fat diet but not under a carbohydrate-rich one; this necessitates the use of techniques able to measure adiposity *in vivo*.

Two additional important observations can be made. Firstly, there was a loose relationship between the HCD and HFD adiposity gains (Pearson’s r=0.482, *P*=0.017, see also individual classification in Figure S4) indicating that being CS does not imply being FS and vice-versa. The second additional observation involves the middle panel of Figure 2. This shows the evolution of the visceral to subcutaneous fat ratio. Across all groups, rats distributed fat more viscerally under HCD than HFD, with the median ratio of 1.82±0.27 at the end of HCD being significantly greater (according to the Wilcoxon signed rank test) than that prior to HCD (1.44±0.29, *P*=0.000) or after HFD (1.53±0.24, *P*=0.000). This does of course have to be considered in the context of absolute fat pad weights (bottom panel of Figure 2), which did increase more under HFD (between MRI2 and Dissection, see Figure 1 for their timing in the context of the study) than under HCD (between MRI1 and MRI2).

As a final point, it should be noted that the HCD, despite having a raw composition very close to that of the chow diet, seems more palatable. There was an acceleration of weight gain during the first week under HCD (on average 4.9 ± 0.3 vs 3.5g ± 0.3 g/day under chow; however this effect was corrected during the second and third weeks (4.0 ± 0.3 then 3.1 ± 0.2 g/day) confirming that “palatability” can be maintained only when the organoleptic properties of the diet are frequently modified as is done for example to maintain hyperphagia under cafeteria feeding.

### Analysis of the relationship between the components of energy expenditure and adiposity gains under both HCD and HFD

We determined how each of Rest-RQ, Act-RQ, activity, Act-cost and RMR measured pre- and post-meal with either HC or HF meals were correlated to adiposity gain under either HCD or HFD (see Figure 1 for how the calorimetry sessions fitted into the overall experimental design). As visible in Figures S2 and S5, the stability of these parameters during the 120 minutes prior to the test meal (08:00 to 10: 00) indicates that this period is a good definition of the pre-meal basal metabolic status, and that the 300 min post-meal period (10: 00 to 15: 00) encompasses most of the meal effect on each parameter.

As described in the Materials and Methods, each metabolic parameter was modeled as a function of adiposity change or absolute adiposity, and whether it was the pre- or post-meal period. This was done separately for each of the HC and HF meals, and each of the adiposity changes under HCD (CR/CS difference) and HFD (FR/FS difference). In the vast majority of cases, no relationships were found: if a particular comparison is not explicitly mentioned here, it can be safely assumed that no significant relationship was observed. However, for readers who may be interested, Table S1 summarizes several of the results obtained on the components of energy expenditure in the simple form of mean±SEM and t-tests for CR vs. CS and FR vs. FS rats after ingestion of the HC or HF test meal and as appropriate during the pre- or post-meal period.

BMR and TEF values did not correlate to adiposity gain during either HCD or HFD. Otherwise stated, rats with a low BMR and/or a low TEF did not exhibit any greater propensity to become obese than rats with a high BMR or TEF. We also observed that the cost of activity increased rapidly in response to ingestion of either the HC or HF meals (see Figures S2 and S5) but also observed no correlation between Act-cost measured before or after the meal and adiposity gain under either diet. This indicates that the energy cost of muscular work indeed varied significantly in relation to the feeding status of the rats, but that differences in the coupling between heat production and muscular work did not influence predisposition to adiposity gain. Considering RQ, no relationship was observed between adiposity gain and Act-RQ, indicating no link between substrate utilization by muscles and propensity to adiposity. Rest-RQ was ^≈^0.80 before meal-delivery and also did not correlate with propensity to adiposity gain.

**Figure 3:**
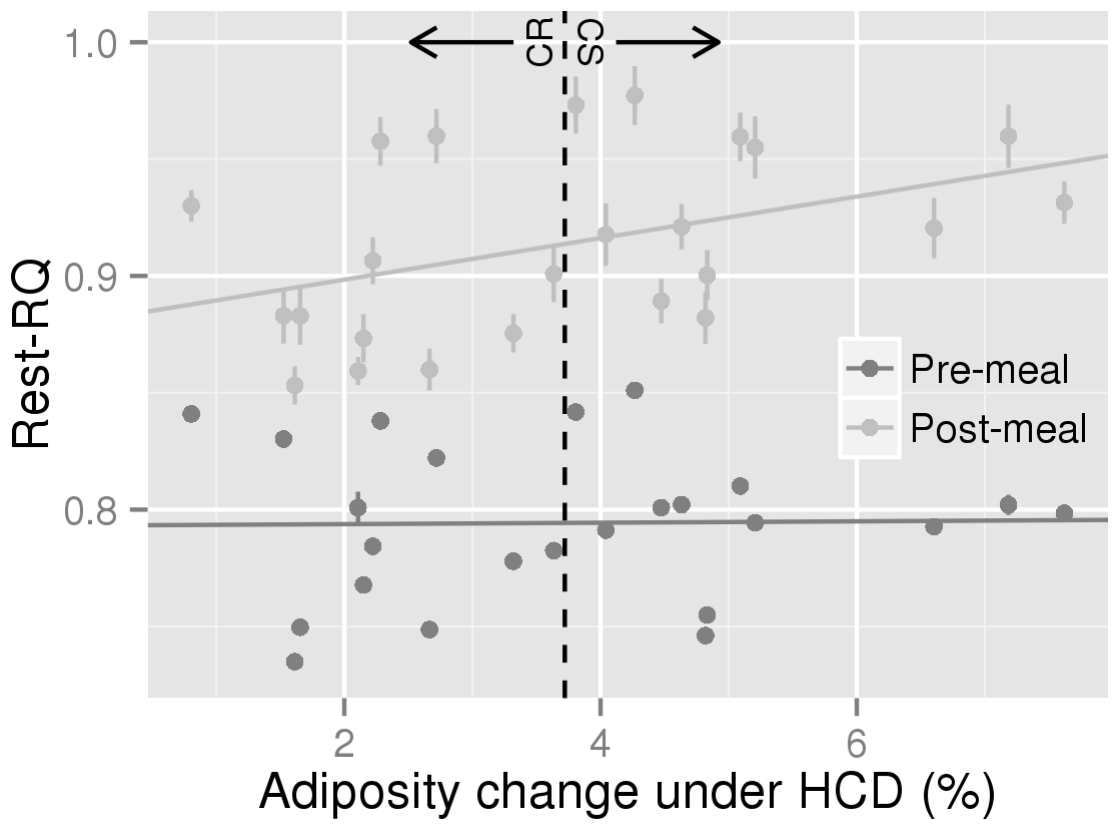
Analysis of the relationship between HCD adiposity gain and Rest-RQ response to a HC meal. Dark grey points (one for each rat) represent mean pre-meal baseline data (-120 to 0 mins), and light grey points (again, one for each rat) the mean post-meal response (0 to 300 mins). The vertical lines on each point represent the individual rat’s SEM. For interest, a broken vertical line is presented separating the CR half of the rats from CS. The pre-meal slope (the fixed effect referred to as “adchg” in the Materials and Methods, describing how pre-meal Rest-RQ is related to adiposity change) is not significantly different from zero (0.0003±0.0041, *P*=0.943). The post-meal slope (the fixed effect referred to as “interaction”, describing how post-meal Rest-RQ response is differently related to adiposity change when compared to the pre-meal slope) is significant (0.0086±0.0026, *P*=0.003) indicating that the higher the post-meal RQ increase, the higher the sensitivity to HCD.

**Figure 4:**
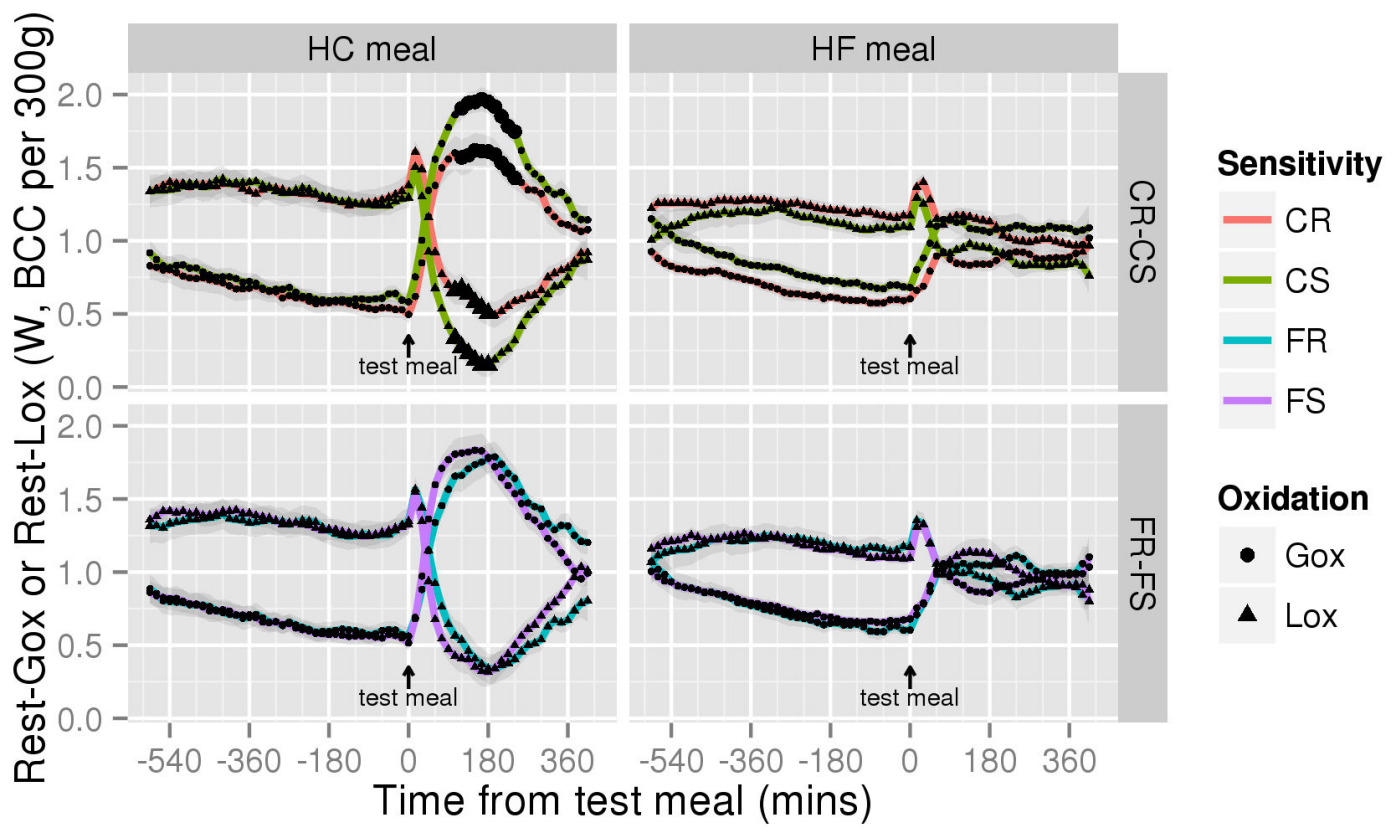
Differences in Rest-Gox and Rest-Lox between CR and CS or FR and FS animals. Rest-Gox (circles) and Rest-Lox (triangles) profiles during HC and HF meal fasting-refeeding procedures between CR (red n=12) and CS (green n=12) or FR (blue n=12) and FS (purple n=12) rats are computed in Watts from resting VO_2_ and VCO_2_ according to the following formulae [10]: Gox=((4.57×VCO_2_)-(3.23×VO_2_))×15.6)/60, Lox=((1.69×VO_2_)-(1.69×VCO_2_))×39.5)/60, with VO_2_ and VCO_2_ in ml/min, 15.6 = kJ/g for glucose, 39.5 = kJ/g for lipids. Division by 60 is to convert Joules per minutes into Joules per second = Watts. BCC = body composition corrected. The underlying data is the same as that for Rest-RQ and REE described in Figure S5, averaged across CS and CR or FR and FS rats. Shaded areas are the group SEM. The meal was given at time=0. The only situation where significant differences appeared was the CR–CS comparison during a HC meal, indicated by the larger circles and triangles (pairwise per-timepoint t-tests *P*<0.01; for Rest-Gox between 120 and 240 mins, for Rest-Lox between 105 and 180 mins inclusive).

In contrast, we observed a significant interaction between the Rest-RQ response to the HC test meal and adiposity changes under HCD (Figure 3, 0.0086±0.0026, *P*=0.003) but not with adiposity changes under HFD (0.0048±0.0025, *P*=0.069, not shown). In general, the Rest-RQ response increased above HCD food quotient (FQ; 0.955) only in CS rats (Figure S5) which means that during this period, the proportion of fat used to fuel energy metabolism was less than the proportion of fat in the meal and was significantly higher than in CR rats between 120 and 180 minutes after ingestion of the meal. Calculation of resting glucose and lipid oxidation (Rest-Gox and Rest-Lox) from Rest-RQ and REE showed that ingestion of the HC test-meal increased Rest-Gox and decreased Rest-Lox significantly more in CS rats (Figure 4, pairwise per-timepoint t-tests *P*<0.01 for both around the 105-240 min period). No differences were observed in the 3 other experimental conditions (Figure 4). Ingestion of the HF meal increased Rest-RQ by only 0.035±0.007 because of its low FQ (0.85).

## Discussion

In this study, the evolution of body composition during periods of both high carbohydrate diet (HCD) and high fat diet (HFD) was longitudinally measured by MRI in male Wistar rats, and assuming thermoregulation was negligible because the rats were housed at 26° C, compared to all the sub-components of TEE, namely REE, and Act-cost and TEF as well as Rest-RQ and Act-RQ measured by indirect calorimetry and extracted by a previously described process of modeling and filtering of raw data [10,12,13]. Taken together, the present observations indicate that in the classic FS obesity-prone rats there is no pre-existing defect in any of the components of EE before the introduction of the HF regimen. In contrast, in CS rats, already under HC diet since weaning (though chow rather than the high quality synthetic HCD used during this experiment), a defective post-prandial substrate partitioning characterized by a larger post-meal increase in Rest-Gox and a larger post-meal inhibition of Rest-Lox can be considered as potentially responsible for the larger adiposity gain. Since this observation was made early in the life of the rats, this metabolic difference can be considered as a cause rather than a consequence of sensitivity to adiposity gain under HCD.

### Weight and adiposity characteristics of the CS rat

Firstly, it needs to be stressed that the HCD used in this study was not designed to induce overfeeding and/or insulin resistance through introduction of a high proportion of sucrose as for example in [19]. It was intended as a baseline replacement of regular chow for better control of nutrient source and delivery, and is based on AIN93 recommendations where most of the carbohydrate is provided as starch [20]. This diet is more palatable than the usual maintenance chow, though as we showed in the results, the palatability effect was short lasting and therefore cannot be considered as the primary driver of adiposity gain under HCD.

Using sequential measurements of body composition by MRI, the present study confirmed a previous one [3] in which we first reported that significant differences in body adiposity between individuals can develop during HCD. The possibility that obesity can develop under HCD has, in general, been rejected for various reasons. The main one is an assumption that carbohydrates are oxidized in proportion to their ingestion, in particular because of the low capacities for glycogen storage and the high cost of lipogenesis from carbohydrates [21,22]. However, considering that only a small amount of fat needs to be deposited each day to induce obesity over the long-term, lipogenesis is not needed (even if possible, in particular under free feeding conditions) as enough fat is already available in a HC diet. Theoretically, all that is needed is for just a bit more of this dietary fat be stored rather than oxidized [22].

Interestingly, an obesity prone (OP, as opposed to resistant, OR) model was recently described in chow fed C57BL/6 mice [54]. At the end of that study, the difference in body weight was larger than the one we observed (25%, estimated from Figure 1 in their article vs. 5.7% in ours), though amongst other experimental differences, prior to body composition analysis their mice were maintained for 6 weeks rather than the 3 weeks of our rats. Like results we previously reported for CR vs. CS rats [3], they observed no differences in daily caloric intake between OR and OP mice. They also focused on analysis of visceral adiposity and reported significantly greater visceral fat relative to body weight in OP mice. Since they did not weigh subcutaneous depots it is not possible to estimate the ratio of subcutaneous to visceral fat accumulation. However, they identified by proteomics that the expression of several proteins contributing to energy metabolism was increased in the visceral adipose tissue of OP mice. These parallel observations, on the two main laboratory rodent models used in human nutrition research, support the validity and the probable universality of the fact that some individuals can gain significantly more weight than others when they ingest low fat or high carbohydrate diets. The question of whether they can become truly obese requires further longer term investigations.

Crucially, differences in sensitivity to adiposity gain under HCD were not very predictive of those under HFD, and vice-versa. Thus individuals needed to be classified not only by their HFD-sensitivity but also by their HCD-sensitivity. An important characteristic of the CR/CS model is that increased adiposity during HCD was difficult to spot from weight measurements as the larger fat deposition in CS rats was not paralleled by an increase in lean mass gain as under HFD, such that overall BW gain was only marginally greater. This is indeed a methodological drawback that imposes the use of *in vivo* adiposity measurement techniques to study the model.

Finally, it must be noted that body fat gain under HCD was characterized in both CR and CS rats by a relatively larger accumulation of fat in the visceral vs. subcutaneous depots; under HFD, while more fat accumulated overall than under HCD, it tended to be more subcutaneous. This suggests that even if overall fat accumulation is lower under HCD than HFD, the adiposity that develops under HCD could be as or more deleterious in the long-term than that which develops in response to HFD [23,24].

### Components of energy expenditure

In general this study found very little link between the various sub-components of TEE and adiposity gain under either HCD or HFD. Among the most important and often suspected parameters involved with resistance/sensitivity to obesity are BMR and TEF. Low BMR values have been suspected to increase the sensitivity to HFD-induced obesity in various animal models as well as in humans [4,25,26], though this is disputed [7,9,27–29]. TEF has also been repeatedly implicated in obesity susceptibility [30–34], but to our knowledge there has been no systematic measurement of TEF in FS rats (or humans) before they developed obesity. Thus, as with BMR, it is not clear if this is really a pre-existing defect as opposed to merely being the result of the obesity and associated metabolic disturbances [15,35]. It was thus important to perform a systematic and well controlled measure of TEF in the rat model before the evolution of weight and adiposity could potentially induce significant metabolic alterations, and in particular for the FS rat, before it had experienced HFD. In addition, in this study we used the only system that to our knowledge (though see 36) is currently able to measure TEF in rats and mice free from noise due to variability in activity intensity; in typical rat metabolic cages, this affects the correct measure of both BEE and post-meal metabolic response from which TEF is calculated. We did not observe correlations between BMR and TEF levels and the propensity to obesity. In our minds, it is not a surprising result since we consider that in highly evolved warm-blooded mammals subjected to large daily to yearly changes in EE with activity (hunting, escaping and migrating) and thermoregulation (sheltered/exposed, summer/winter), the mechanisms regulating energy homeostasis have necessarily adapted to compensate for the large variations in EE necessary for coping with the environment, a capacity also demonstrated in obese rats [37,38].

Differences in activity have been reported between FR and FS rats or between lean and obese rats [6,39,40]. Fidgeting is also suspected to account for a large part of the daily EE due to activity and to participate in resistance to obesity [29]. In the present study we observed no differences in activity, though in order to properly measure BMR and TEF the rats were not under usual free feeding conditions and therefore this observation does not preclude possible differences under more normal living conditions. More significant was the observation that there were no differences in the cost of activity between resistant and sensitive rats in the fasted as well as in the fed state. Numerous studies have investigated the possibility that the amount of energy lost as a result of uncoupling between ATP generation and mechanical work may be reduced in obesity prone subjects, particularly in muscles which express UCP3 and where uncoupling appears larger than in other tissues [41]. In previous studies most of the information was derived from *in*- or *ex vivo* measurement of resting muscles [42–46]. In the present study, we report the first measure of variability in the cost of muscular effort in freely moving rats by a non-invasive method based on the short-term analysis of the relationship between VO_2_-VCO_2_ and the intensity of activity by the Kalman method [12]. This can be considered as an important advance as it allows observation, in freely moving animals, of the efficiency of muscular work, analysis of changes between groups and within subjects and in the case of this study the transition from the fasted to the fed state. The main observation we made was that despite the fact that cost of activity indeed varied largely over time in relation to the fasted/fed status of the rats, individual differences in the cost of activity were not found to correlate with adiposity gain under HC or HF feeding. This means that uncoupling in working muscles is larger when the animal is fed, but does not depend on the substrate used by muscles, and that differences in uncoupling are not among the metabolic processes that appear to be linked to predisposition to obesity. Further studies are necessary to confirm this first report, but if substantiated, this observation may be important in a context where a large quantity of resources are being invested in the hypothesis that increased uncoupling in muscles may be a means to fight against obesity.

Together with the cost of activity, the processing of the short-term changes in activity and VO_2_-VCO_2_ allowed us to study Act-RQ, meaning to follow along the time the fuel mix used by working muscles. The currently available data estimating lipid oxidation by muscles are often contradictory. For example, Dourmaskin and col. reported that before becoming obese, FS rats suffer metabolic disturbances favoring fat storage and in particular a decline in lipid transport and capacity for lipid oxidation in muscles [47], while Commerford, Pagliassotti et al. reported differences in the rate of lipid oxidation and FFA cycling between FR and FS rats [48]. Here, we observed that Act-RQ was always fairly close and usually a little higher than Rest-RQ, indicating that the fuel mix used by working muscles for spontaneous activity (as opposed to forced exercise) is close to the fuel mix used by the rest of the body. Interestingly, the increase in Act-RQ induced by ingestion of the test-meal was as rapid as the one measured on whole body RQ (Figures S2 and S5). As with Act-cost, we did not observe any differences between sensitive and resistant rats. This suggests that before obesity develops, differences in the fuel mix used to supply muscle effort in relation to spontaneous activity are also not related to obesity sensitivity. However, this result is the first report of muscle fuel mix measurement in freely moving rats and requires further studies to be confirmed.

In contrast, Rest-RQ, meaning RQ computed from resting VO_2_ and VCO_2_ values, increased more in response to HC feeding in CS than in CR rats. Converted into rates of glucose and lipid oxidation, this difference reflected the fact that the post-meal increase in Rest-Gox and decrease in Rest-Lox were of larger amplitude in CS rats. Accordingly, we previously reported that under free feeding there is a positive correlation between adiposity gain and 24h RQ in rats fed HCD [3]. Taken together, these results indicate that the percentage of fat in the fuel mix used to supply whole body resting energy metabolism is smaller (more smaller than in CR rats) than the proportion of fat in the diet, suggesting a possible mechanism for CS rats to fix more fat than CR ones under HCD. Whether fat progressively accumulates as a result of direct storage of dietary fat and/or after stimulation of lipogenesis from glucose remains to be determined. In both this study and the previous one [3], RQ never increased above 1, indicating no net lipogenesis.

It has been suggested that nutrient partitioning or metabolic flexibility (meaning the capacity of the organism to adapt fuel oxidation to food composition) may be responsible for establishing different adiposity levels in the long-term [49–51]. This was described as a “metabolic inflexibility”, meaning the inability to switch from fat to carbohydrate oxidation in response to feeding or insulin clamp [50–52]. Our results rather suggest a “metabolic hyperflexibility” in CS rats. This difference may be due to (i) observation of CS rats, not just FS ones and (ii) making measurements before rather than after obesity had developed. Follow up of the response to HC and HF meals along with adaptation to a HFD should be performed to verify how meal-induced RQ and RMR changes evolve and discriminate between differences that precede and those that are the consequences of HFD.

## Conclusion

This study firmly establishes recent observations that significant differences in body fat accumulation develop under HCD in rats. The sensitivity to HCD was correlated to that under HFD, but not strongly, indicating that HCD- and HFD-sensitivities do not necessarily share common underlying metabolic defects. This study also indicates that FS rats do not exhibit any defect in any components of TEE and RQ before being submitted to HFD; to our knowledge this leaves for now circulating triglycerides after a high-fat preload as the only good predictor in rats of later sensitivity to HFD [53]. In contrast, we observed that in fed CS rats adapted to a HC diet, lipid oxidation is lower and glucose oxidation larger than in CR rats. This CS model appears to be a new rodent model of sensitivity to obesity that may help to explore other potential mechanisms involved in the predisposition to obesity in humans and in the dissociation sometimes observed with fat intake.

## Supporting Information

Figure S1 Pre- and post-selection adiposity. Adiposity measured during the initial MRI session showing the greater initial adiposities of the rats selected for inclusion in the study.
(TIF)

Figure S2 Example of data recording and processing in an individual rat. The time frame is reduced to from 0: 00 to 17: 00 for clarity. Top: original raw data as recorded by the gas analyzers and activity monitors. RQ is the ratio of VCO_2_: VO_2_. Middle: data of the top panel processed by Kalman filtering. VO_2_ and VCO_2_ are transformed into metabolic rate (TMR, in watts) according to Weir formula =((VO_2_×16.3)+(VCO_2_×4.57))/60. Modeling and processing of the changes in VO_2_ and VCO_2_ in relation to Act gives computation of RMR. RQ, Resting RQ (Rest-RQ) and Activity RQ (Act-RQ) are computed from resting and activity VO_2_ and VCO_2_ respectively. Bottom: metabolic cost of activity computed by the Kalman filter as the amplitude of changes in VO_2_ and VCO_2_ relative to intensity of activity. Vertical grey bar at 10: 00 shows the time interval during which data acquisition was frozen to introduce the test-meal.
(PDF)

Figure S3 Individual body weight changes during the study. The data are sorted into rat adiposity sensitivity or resistance groups as defined in Figure 2.
(TIF)

Figure S4 Rankings of individual rat sensitivities to adiposity gain under HCD and HFD. (TIF)

Figure S5 Components of energy expenditure. The six panel groups show meal response metabolic cage data collected during the HCD period (time frame reduced to from 10hrs before to 6 hours after meal onset for clarity). X axis labeled in minutes vs. meal onset. Rest-RQ, Act-RQ, Activity, Act-cost and RMR (BCC = body composition corrected) are all defined as described in the Materials and Methods. The data are sorted into rat adiposity sensitivity or resistance groups as defined in Figure 2, plus according to whether the session involved refeeding with a HC or HF meal (given at time=0). Each thin grey line represents a single rat calorimetry session; the thick black line is the mean, and the grey shadow extending either side the group SEM. Each rat experienced two sessions, one for each of the HC and HF meals, and in each session all the data necessary to derive these six metabolic parameters was collected. The broken vertical lines (from left to right at -120, 0 and 300 mins) represent the bounds of data selected for linear mixed modeling statistics. For RQ graphs, the broken horizontal lines show the food quotient (FQ) of the ingested meal.
(TIF)

Table S1 Categorical comparisons of various body composition and metabolic parameters. Results are in the simple form of mean±SEM and t-tests for CR vs. CS and FR vs. FS rats after ingestion of the HC or HF test meal and as appropriate during the pre- or post-meal period. BW = body weight, FFM = fat-free mass, FM = fat mass, BMR = basal metabolic rate, TEF = thermic effect of feeding, AUC = area under the curve (relative to basal data), W/AUA = Watts per Arbitrary Unit of Activity, NS = not significant (p>0.01). BMR and other basal metabolic data were acquired during the 120 minute pre-meal period. The post-meal period covered the 300 minutes following meal provision.
(DOCX)
