# Supplementary figures and images for: The Carbohydrate Sensitive Rat as a Model of Obesity
Source: PLoS One. 2013 Jul 30;8(7):e68436. doi: 10.1371/journal.pone.0068436 (PMC3728328; doi:10.1371/journal.pone.0068436)

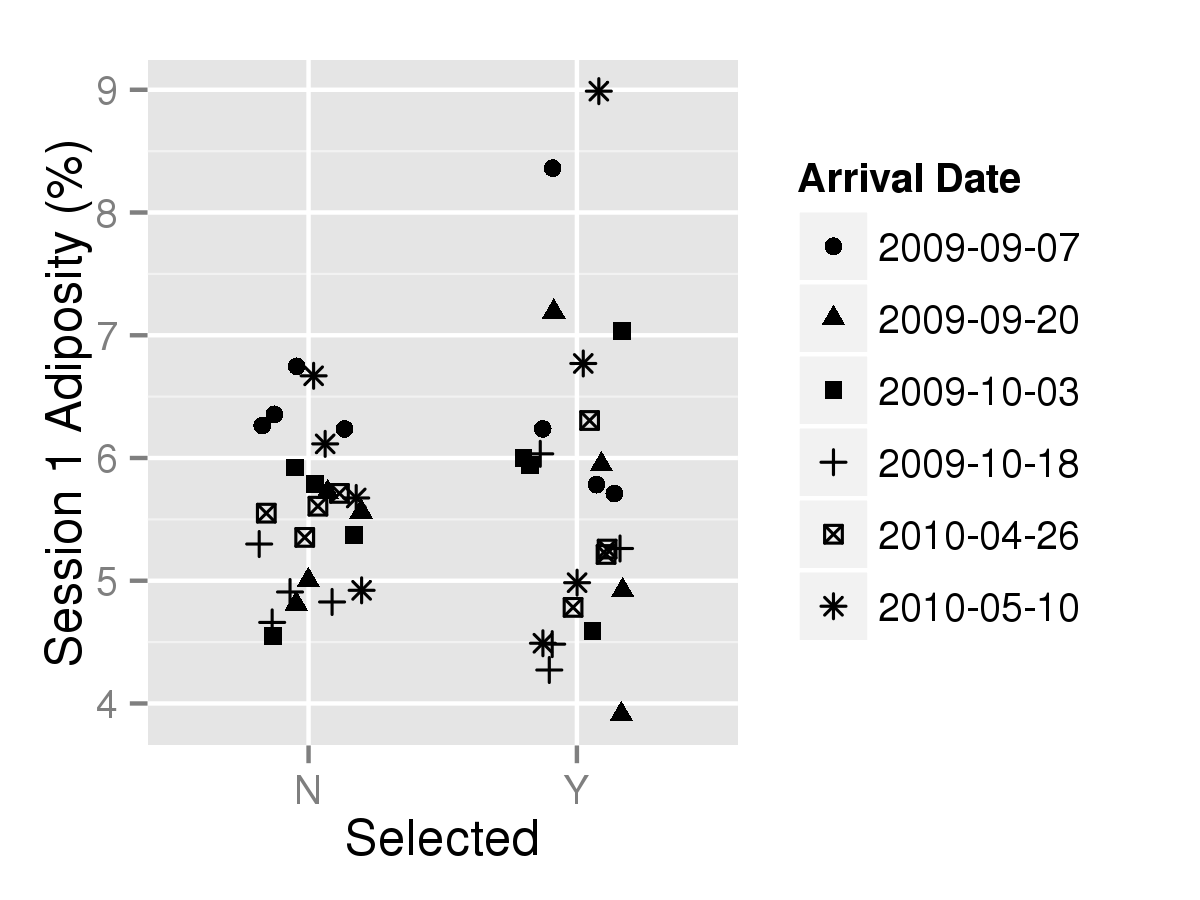

Supplement: Figure S1 — Adiposity measured during the initial MRI session showing the greater initial adiposities of the rats selected for inclusion in the study. (TIF) [file pone.0068436.s001.tif]

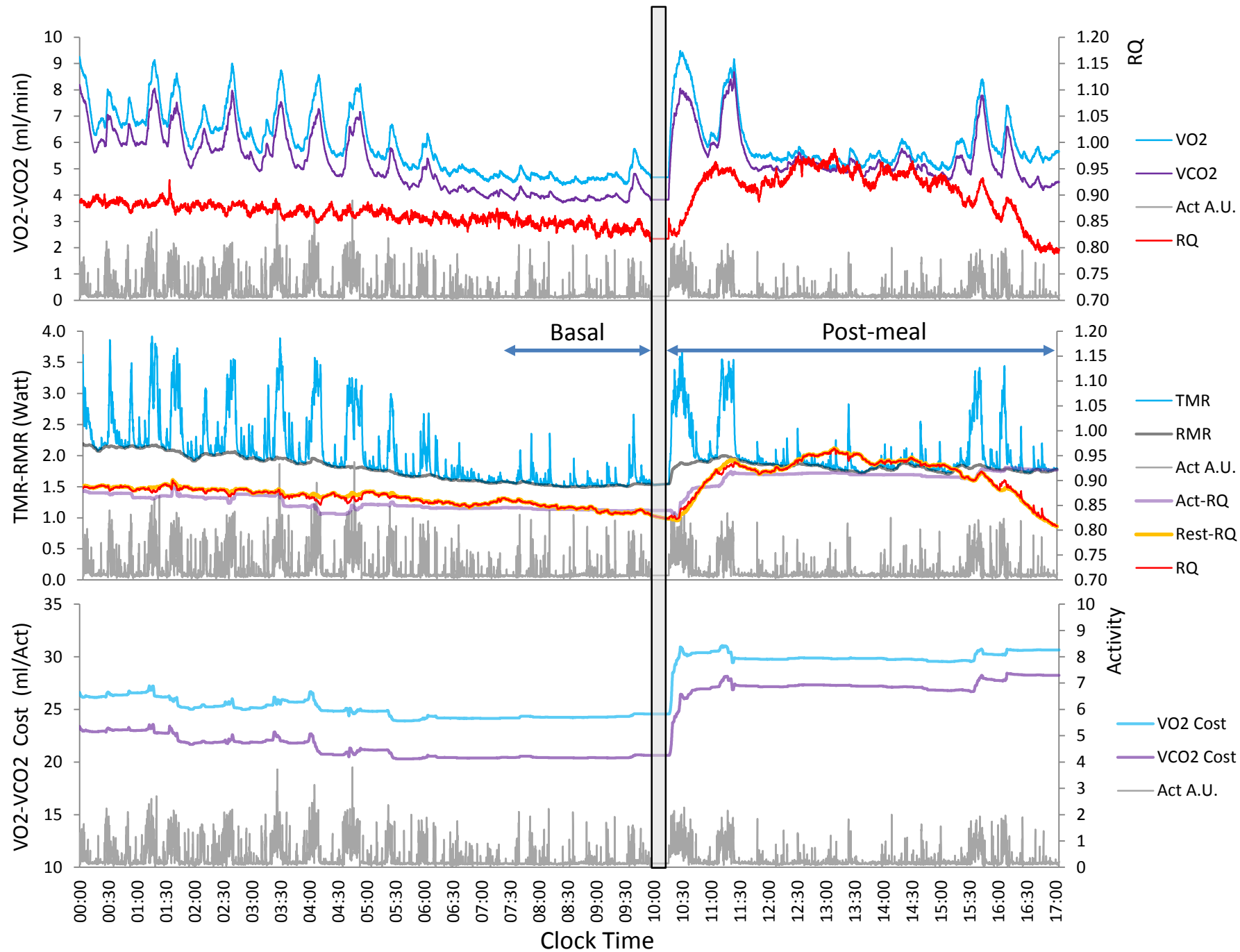

Supplement: Figure S2 — The time frame is reduced to from 0: 00 to 17: 00 for clarity. Top: original raw data as recorded by the gas analyzers and activity monitors. RQ is the ratio of VCO2: VO2. Middle: data of the top panel processed by Kalman filtering. VO2 and VCO2 are transformed into metabolic rate (TMR, in watts) according to Weir formula =((VO2×16.3)+(VCO2×4.57))/60. Modeling and processing of the changes in VO2 and VCO2 in relation to Act gives computation of RMR. RQ, Resting RQ (Rest-RQ) and Activity RQ (Act-RQ) are computed from resting and activity VO2 and VCO2 respectively. Bottom: metabolic cost of activity computed by the Kalman filter as the amplitude of changes in VO2 and VCO2 relative to intensity of activity. Vertical grey bar at 10: 00 shows the time interval during which data acquisition was frozen to introduce the test-meal. (PDF) [file pone.0068436.s002.pdf]

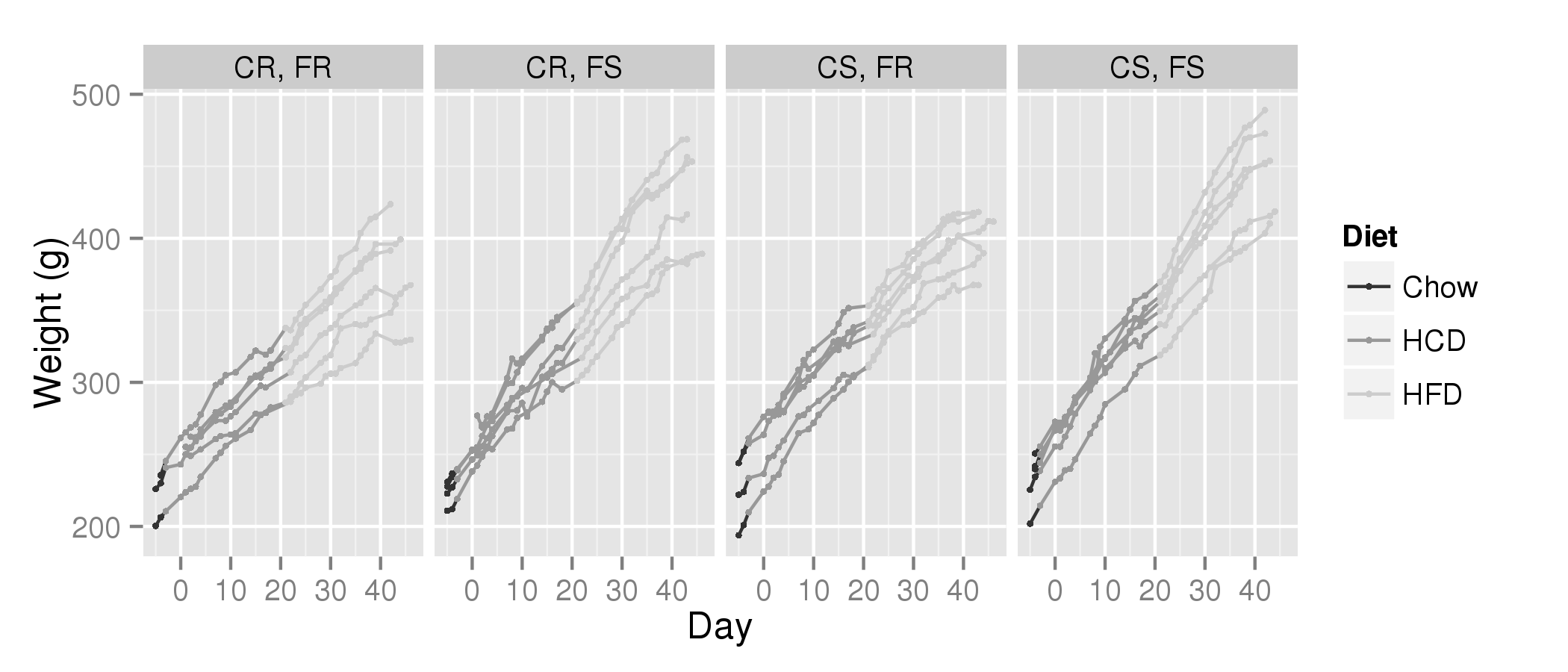

Supplement: Figure S3 — The data are sorted into rat adiposity sensitivity or resistance groups as defined in Figure 2. (TIF) [file pone.0068436.s003.tif]

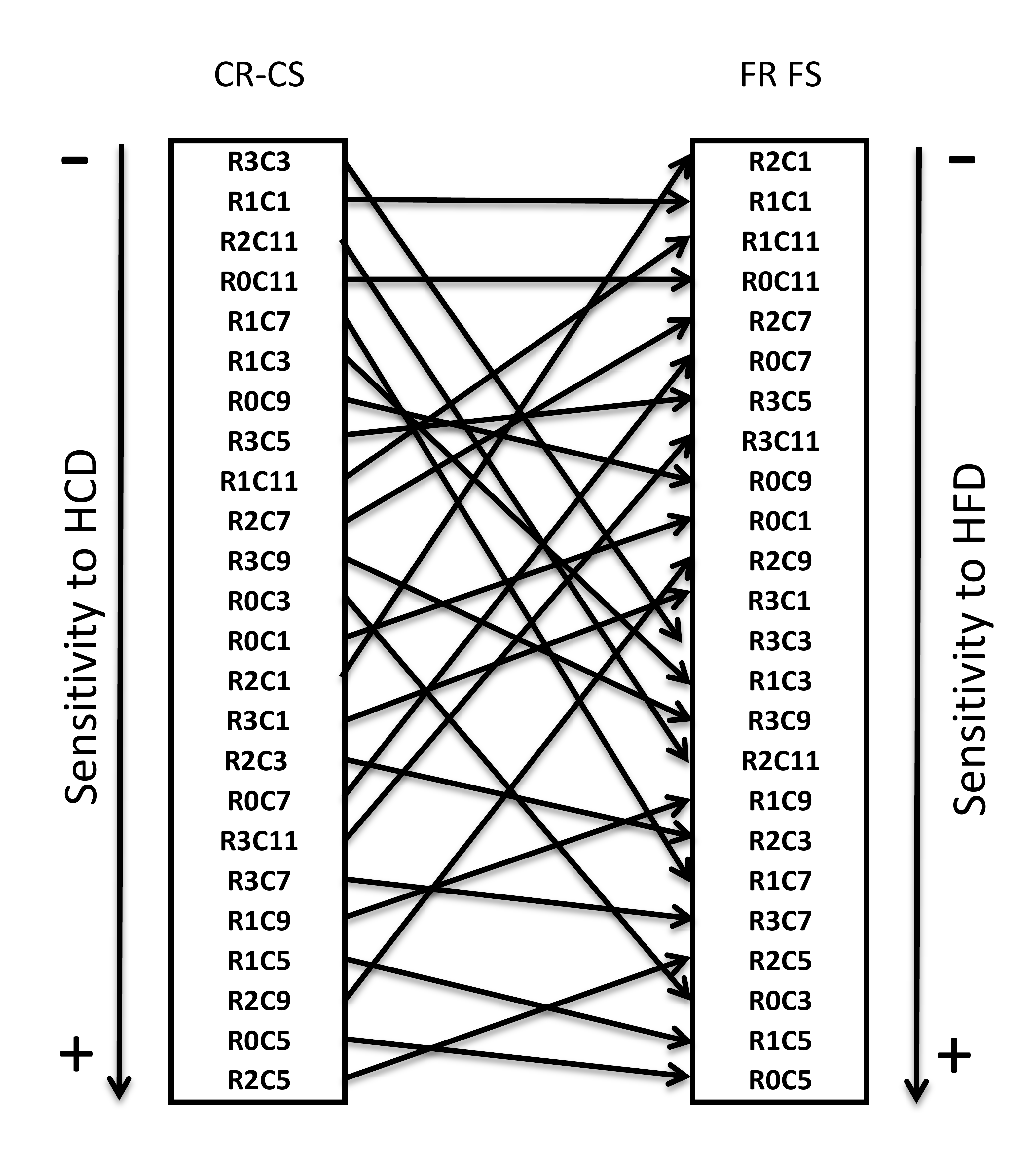

Supplement: Figure S4 — (TIF) [file pone.0068436.s004.tif]

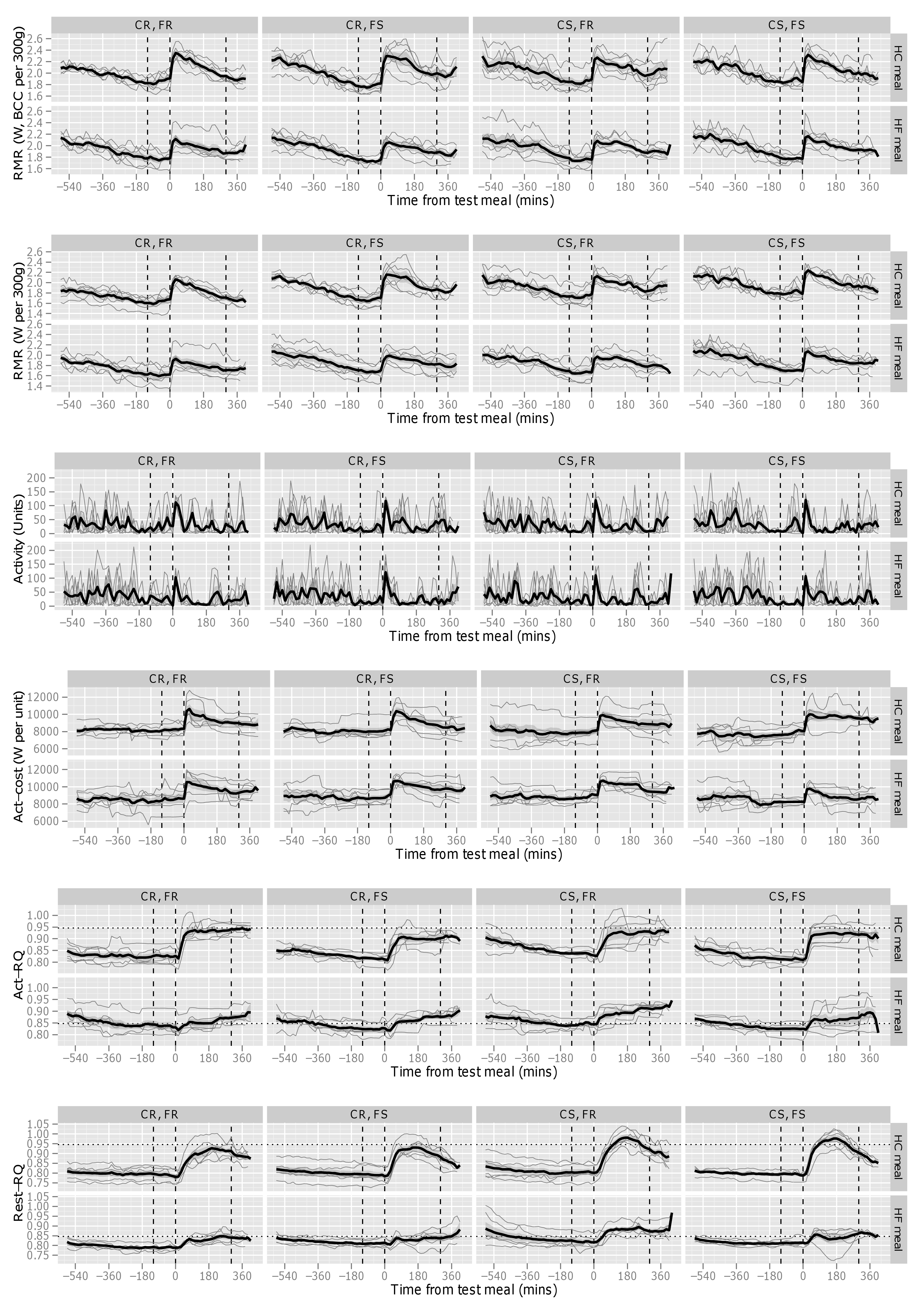

Supplement: Figure S5 — The six panel groups show meal response metabolic cage data collected during the HCD period (time frame reduced to from 10hrs before to 6 hours after meal onset for clarity). X axis labeled in minutes vs. meal onset. Rest-RQ, Act-RQ, Activity, Act-cost and RMR (BCC = body composition corrected) are all defined as described in the Materials and Methods. The data are sorted into rat adiposity sensitivity or resistance groups as defined in Figure 2, plus according to whether the session involved refeeding with a HC or HF meal (given at time=0). Each thin grey line represents a single rat calorimetry session; the thick black line is the mean, and the grey shadow extending either side the group SEM. Each rat experienced two sessions, one for each of the HC and HF meals, and in each session all the data necessary to derive these six metabolic parameters was collected. The broken vertical lines (from left to right at -120, 0 and 300 mins) represent the bounds of data selected for linear mixed modeling statistics. For RQ graphs, the broken horizontal lines show the food quotient (FQ) of the ingested meal. (TIF) [file pone.0068436.s005.tif]
