# Supplementary material for: The Carbohydrate Sensitive Rat as a Model of Obesity
Source: PLoS One. 2013 Jul 30;8(7):e68436. doi: 10.1371/journal.pone.0068436 (PMC3728328; doi:10.1371/journal.pone.0068436)
Supplement: Table S1 — Results are in the simple form of mean±SEM and t-tests for CR vs. CS and FR vs. FS rats after ingestion of the HC or HF test meal and as appropriate during the pre- or post-meal period. BW = body weight, FFM = fat-free mass, FM = fat mass, BMR = basal metabolic rate, TEF = thermic effect of feeding, AUC = area under the curve (relative to basal data), W/AUA = Watts per Arbitrary Unit of Activity, NS = not significant (p>0.01). BMR and other basal metabolic data were acquired during the 120 minute pre-meal period. The post-meal period covered the 300 minutes following meal provision. (DOCX) [file pone.0068436.s006.docx]

|  |  | **CR vs. CS** | |  | |  |  | **FR vs. FS** | |  | |  |
| --- | --- | --- | --- | --- | --- | --- | --- | --- | --- | --- | --- | --- |
|  |  | **CR (n=12)** | | **CS (n=12)** | | **t-test** |  | **FR (n=12)** | | **FS (n=12)** | | **t-test** |
|  |  | **mean** | **SE** | **mean** | **SE** | **p-value** |  | **mean** | **SE** | **mean** | **SE** | **p-value** |
| **HC meal** |  |  |  |  |  |  |  |  |  |  |  |  |
| Body composition (g) | BW | 291.1 | 5.7 | 315.5 | 6.8 | NS |  | 293.1 | 7.4 | 313.5 | 5.7 | NS |
|  | FFM | 268.8 | 4.6 | 279.9 | 4.3 | NS |  | 268.2 | 5.1 | 280.5 | 3.5 | NS |
|  | FFM + 0.2 × FM | 272.6 | 4.8 | 285.9 | 4.7 | NS |  | 272.6 | 5.5 | 285.9 | 3.9 | NS |
| BMR (W) | Absolute | 1.655 | 0.036 | 1.770 | 0.039 | NS |  | 1.685 | 0.042 | 1.740 | 0.038 | NS |
|  | per 300g BW | 1.709 | 0.037 | 1.687 | 0.033 | NS |  | 1.729 | 0.037 | 1.666 | 0.031 | NS |
|  | per 300g FFM | 1.848 | 0.035 | 1.899 | 0.038 | NS |  | 1.886 | 0.038 | 1.861 | 0.035 | NS |
|  | per 300g FFM + 0.2 × FM | 1.823 | 0.034 | 1.859 | 0.036 | NS |  | 1.856 | 0.037 | 1.826 | 0.034 | NS |
| Other basal data | Rest-RQ | 0.790 | 0.011 | 0.799 | 0.009 | NS |  | 0.797 | 0.010 | 0.792 | 0.009 | NS |
|  | Act-RQ | 0.821 | 0.008 | 0.824 | 0.007 | NS |  | 0.831 | 0.008 | 0.814 | 0.006 | NS |
|  | Act-cost (W/AUA) | 8.164 | 0.237 | 7.931 | 0.235 | NS |  | 8.103 | 0.245 | 7.993 | 0.231 | NS |
| Post-meal data (AUC) | TEF (kJ) | 5.196 | 0.524 | 4.479 | 0.467 | NS |  | 4.392 | 0.445 | 5.283 | 0.531 | NS |
|  | Rest-RQ | 2.107 | 0.155 | 2.669 | 0.112 | 0.008 |  | 2.304 | 0.166 | 2.472 | 0.148 | NS |
|  | Act-RQ | 1.715 | 0.206 | 1.764 | 0.234 | NS |  | 1.721 | 0.235 | 1.758 | 0.206 | NS |
|  | Act-cost (W/AUA) | 24.37 | 4.94 | 31.12 | 4.03 | NS |  | 25.22 | 4.98 | 30.26 | 4.10 | NS |
| **HF meal** |  |  |  |  |  |  |  |  |  |  |  |  |
| Body composition (g) | BW | 298.5 | 7.6 | 317.4 | 6.8 | NS |  | 300.6 | 7.5 | 315.3 | 7.4 | NS |
|  | FFM | 278.1 | 6.1 | 280.0 | 5.7 | NS |  | 274.3 | 5.4 | 283.7 | 6.0 | NS |
|  | FFM + 0.2 × FM | 282.3 | 6.3 | 286.1 | 5.9 | NS |  | 279.2 | 5.7 | 289.2 | 6.2 | NS |
| BMR (W) | Absolute | 1.652 | 0.042 | 1.680 | 0.038 | NS |  | 1.639 | 0.042 | 1.693 | 0.037 | NS |
|  | per 300g BW | 1.664 | 0.035 | 1.590 | 0.022 | NS |  | 1.641 | 0.040 | 1.613 | 0.018 | NS |
|  | per 300g FFM | 1.783 | 0.025 | 1.802 | 0.030 | NS |  | 1.794 | 0.034 | 1.791 | 0.020 | NS |
|  | per 300g FFM + 0.2 × FM | 1.756 | 0.025 | 1.763 | 0.028 | NS |  | 1.762 | 0.033 | 1.757 | 0.019 | NS |
| Other basal data | Rest-RQ | 0.801 | 0.006 | 0.816 | 0.007 | NS |  | 0.804 | 0.008 | 0.812 | 0.005 | NS |
|  | Act-RQ | 0.831 | 0.009 | 0.836 | 0.007 | NS |  | 0.841 | 0.008 | 0.825 | 0.007 | NS |
|  | Act-cost (W/AUA) | 8.685 | 0.282 | 8.564 | 0.160 | NS |  | 8.731 | 0.266 | 8.517 | 0.181 | NS |
| Post-meal data (AUC) | TEF (kJ) | 3.628 | 0.465 | 4.194 | 0.349 | NS |  | 3.825 | 0.460 | 3.998 | 0.374 | NS |
|  | Rest-RQ | 0.574 | 0.147 | 0.826 | 0.256 | NS |  | 0.934 | 0.170 | 0.466 | 0.226 | NS |
|  | Act-RQ | 0.511 | 0.160 | 0.820 | 0.205 | NS |  | 0.627 | 0.158 | 0.705 | 0.216 | NS |
|  | Act-cost (W/AUA) | 25.73 | 3.27 | 20.30 | 4.00 | NS |  | 25.55 | 2.37 | 20.48 | 4.60 | NS |
